# Binder-free Sn–Si heterostructure films for high capacity Li-ion batteries

**DOI:** 10.1039/c7ra13489d

**Published:** 2018-05-08

**Authors:** M. J. Loveridge, R. Malik, S. Paul, K. N. Manjunatha, S. Gallanti, C. Tan, M. Lain, A. J. Roberts, R. Bhagat

**Affiliations:** WMG, Warwick University Coventry CV4 7AL UK M.Loveridge@warwick.ac.uk; EMTERC, De Montfort University Leicester LE19BH UK

## Abstract

This study fabricated and demonstrated a functional, stable electrode structure for a high capacity Li-ion battery (LIB) anode. Effective performance is assessed in terms of reversible lithiation for a significant number of charge–discharge cycles to 80% of initial capacity. The materials selected for this study are silicon and tin and are co-deposited using an advanced manufacturing technique (plasma-enhanced chemical vapour deposition), shown to be a scalable process that can facilitate film growth on 3D substrates. Uniform and hybrid crystalline–amorphous Si nanowire (SiNW) growth is achieved *via* a vapour–liquid–solid mechanism using a Sn metal catalyst. SiNWs of less than 300 nm diameter are known to be less susceptible to fracture and when grown this way have direct electrical conductivity to the current collector, with sufficient room for expansion. Electrochemical characterisation shows stable cycling at capacities of 1400 mA h g^−1^ (>4 × the capacity limit of graphite). This hybrid system demonstrates promising electrochemical performance, can be grown at large scale and has also been successfully grown on flexible carbon paper current collectors. These findings will have impact on the development of flexible batteries and wearable energy storage.

## Abstract 2

This study fabricated and demonstrated a functional, stable electrode structure for a high capacity Li-ion battery (LIB) anode.

## Graphical abstract

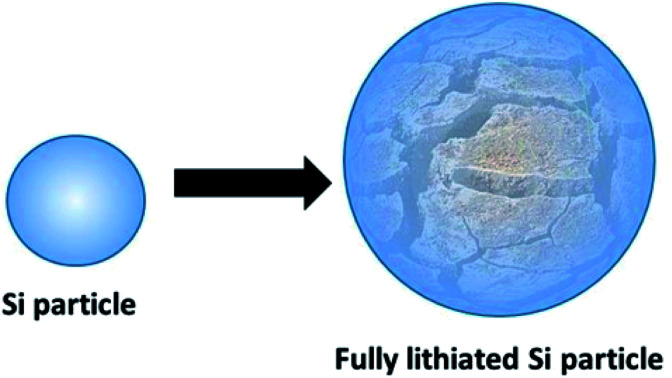

## 1 Introduction

The US Dept. of Energy cost target for all electric vehicles is $125 per kWh of usable energy but the current cost of commercial batteries is $400–500 per kWh.^1^ Cost reductions are possible through evolution away from conventional electrode fabrication practices to alternative battery manufacturing schemes.^2–4^ This focuses on processes that do not require multi-stage dispersion, indirect materials (solvents that do not end up in the product), other chemical processes, drying and solvent recovery. At the same time the energy storage community remains heavily engaged in trying to better understand the implications of electron and ion transport within electrode architectures and their influence on electrochemical performance – this is made challenging when there is little control over the electrode structure.^5^ With a better understanding, it is possible to design efficient battery components to overcome such transport issues. Development of electrodes that can retain their microstructure as they are charged and discharged over thousands of cycles is a critical element in creating batteries that will overcome range anxiety in electrified vehicles.^6^

Non-conventional manufacturing methods have been explored to create battery electrodes^1^ yet none of them have inspired commercial uptake into mainstream manufacturing methodology. These include: (1) solvent-based electrostatic spray deposition,^7^ (2) spray painting/electrostatic spraying, (3) dry electrode manufacturing *e.g.* pulsed laser and sputter deposition,^8^ (4) magnetron sputtering.^9^ Better understanding of the process itself has added valuable insight into directly improving the process for fabricating Li-ion battery electrodes, especially the means by which materials are uniformly deposited.^10^

**Fig. 1:**
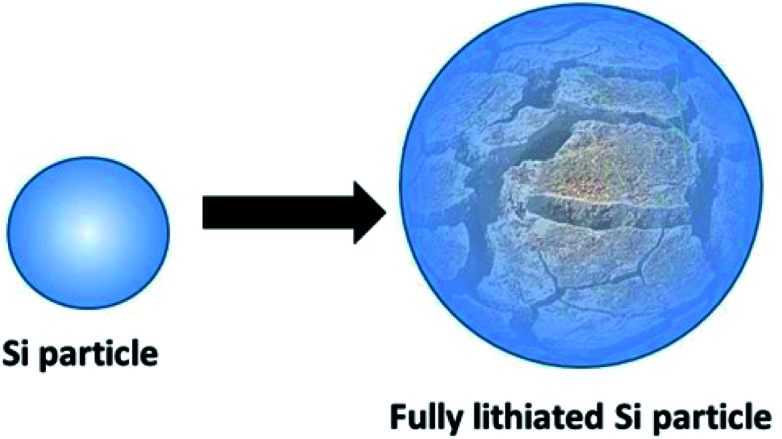
Illustration of lithiation-induced Si particle fracture.

Intense and widespread research efforts into new materials for Li-ion batteries (LIBs) have focused a lot of interest on the group IV elements (Si and Sn primarily).^11,12^ Si and Sn are the two most studied materials both individually and as an alloy negative electrode materials and both have made some progress towards entering the commercial arena. Cost analysis for these materials shows Sn be clearly the more economical on a unit cost per Ah basis (graphite : Si : Sn = 0.81 : 0.08 : 1.51 c per Ah) with Si being the second most abundant element on earth. These elements have high lithiation capacities of 3579 and 994 mA h g^−1^ respectively, compared with mature graphite anodes of limited capacity of 372 mA h g^−1^.^11^ However, the group IV metals and metalloids interact with Li ions differently^13^ (compared with graphite) and instead of intercalating, they alloy with Li^+^ to the extent of incurring a deleterious volume expansion upon reversible cycling reactions.^14,15^ This volume increase can reach up to 300% when approaching the maximum capacity of Si and such expansion in turn leads to particle pulverisation (Fig. 1), electrode cracking and the progressive growth of a solid-electrolyte interface (SEI).^16–18^

In contrast to the cathode electrolyte interface (CEI), as is common with materials such as lithium iron phosphate, and the stable SEI formed on graphite, with silicon the SEI is unstable^19^ and continues to grow.^20^ This represents a cumulative, irreversible loss of the Li “inventory” as well as a progressive increase in surface resistance on the Si particles, which results in pronounced and premature capacity decrease as a function of cycle number. This has precluded anodes based on Si from serious attempts at incorporating such materials into batteries for vehicle electrification. There have been an extensive number of studies on both Si and Sn as separate active materials for Li-ion batteries^21–24^ but it is not the purpose of this article to incorporate an extensive review of these studies – here we focus on the hybridisation of these elements in a non-conventional manufacturing approach.

More recently there have been attempts at hybridising Si and Sn within the same electrode, *e.g.* an investigation using Sn nanoparticles as an effective, conductive additive for Si-based anodes in Li-ion half-cells.^25–27^ The first study claimed that the presence of the Sn (as low as 2%) dramatically improves the electrode's performance in terms of both charge capacity and cycling stability.^26^ It proposed to have achieved this by being uniformly dispersed in the Si network but also reducing the electrical resistance of the electrode structure as a whole. As such, Mangolini *et al.* the synergistic effects between the materials lead to batteries that exceed the performance of each of the two components alone.^25^ This is attributed to the high electrical conductivity and good reversibly energy storage capacity of Sn. Other researchers have looked at silicon–tin hybrid anode systems for solid-state Li-ion batteries achieving reversible capacities up to 700 mA h g^−1^.^28^ Combining hybridised “yin-yang” silicon–tin porous nanocomposites with graphene has also been used as an approach for generating low-cost and low energy consumption materials with promising electrochemical performance.^29^

When considering silicon as the predominant anode active material, the one-dimensional aspect of silicon nanowires (SiNWs) has received widespread attention^30,31^ but will not be discussed at length here. It reported to be structurally beneficial as an anode active material as it: (1) allows sufficient space between collections of nanowires to accommodate the volume changes brought about by lithiation, and (2) allows axial/radial stress relaxation of the nanowires.^32^ This relaxation is thought to alleviate any progressive pulverisation that is commonly observed in the bulk and thick film Si structures during operational cycling.^33,34^ Metal-assisted vapour–liquid–solid (VLS) mechanism is a widely used approach to obtain anisotropic 1D nanowires owing to its simplicity and versatility with regards to semiconductor nanowires.^35,36^ This method represents an alternative manufacturing route beyond conventional composite electrode fabrication methodology, and could constitute an economically viable, less energy-intense production route within energy storage manufacturing.^37^

**Fig. 2:**
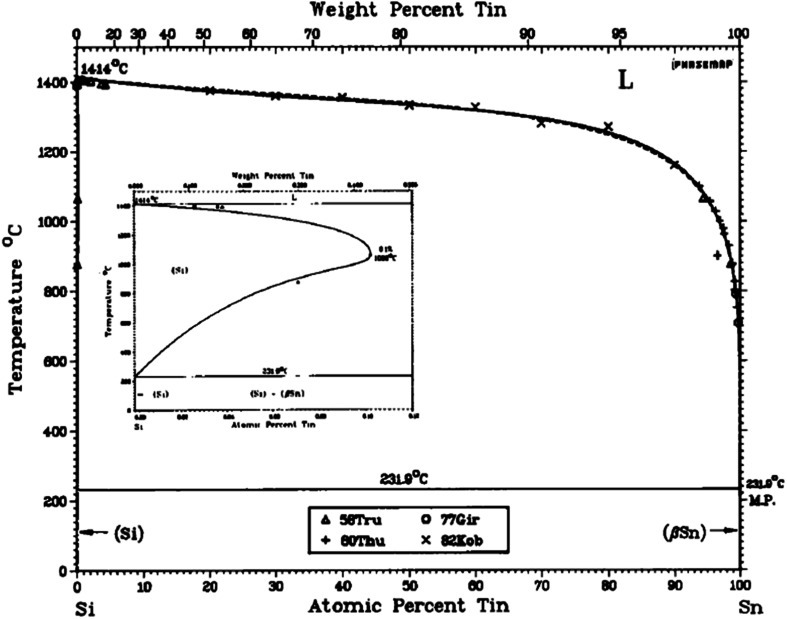
The Si–Sn equilibrium phase diagram^40^.

The crystalline properties of nanowires grown this way is considered to be of good quality and sometimes referred to as “defect-free”, with the exception of multiple twin defects that can cut across the nanowires (described further on in this paper).^35^ Si is a commonly used alloying element in several grades of aluminium and steel^38^ but does not alloy to a high degree with Sn since the solubility of Sn in Si is very low (≈5 × 10^19^ cm^−3^)^39^ as illustrated by the binary phase diagram for the silicon-tin alloy system (see Fig. 2).

Consistent with the Hume-Rothery limits of binary solubility, Sn and Si have atomic radii differences significantly >15% and as a result have limited solubility in each other. This is despite their other favourable common properties in this respect, namely crystal structure, valence and electronegativity.^41^ It is this limited solubility with Si that makes Sn a good catalyst in this instance as it tends to generate atomically sharp heterostructures.^42^ With this in mind, this study essentially integrates a Sn granular thin film with nano-crystalline and amorphous phase Si nanowires (SiNWs), to generate a degradation-resistant, high capacity anode system. In order to synthesise the Si nanowires use of Radio-Frequency Plasma Enhanced Chemical Vapour Deposition (RF-PECVD) technique for the nanowire growth that incorporates vapour–liquid–solid (VLS) catalyst mediated growth process, which is described as a “bottom–up” method.^43^ In this process, the substrate coated with Sn as a catalyst is heated in the presence of a hydrogen and silane precursor gas, which preferentially absorbs Si atoms and precipitated out of the Sn catalyst. Upon dissolution into the Sn droplet, Si atoms form a liquid eutectic alloy with the underlying Sn catalyst. Eventually – with continuous flow of SiH_4_ – the alloy becomes supersaturated whereby the nucleation barrier is surpassed and Si precipitates at the liquid–solid interface, minimising the free energy of the system.^44^ Because the mechanism consists of adsorption, dissolution, diffusion and precipitation in the liquid phase – these are thermodynamic processes that work towards equilibrium – one can refer to the Sn–Si equilibrium phase diagram in Fig. 2 to understand catalyst mediated growth of solid SiNWs from a liquid catalyst.

**Fig. 3:**
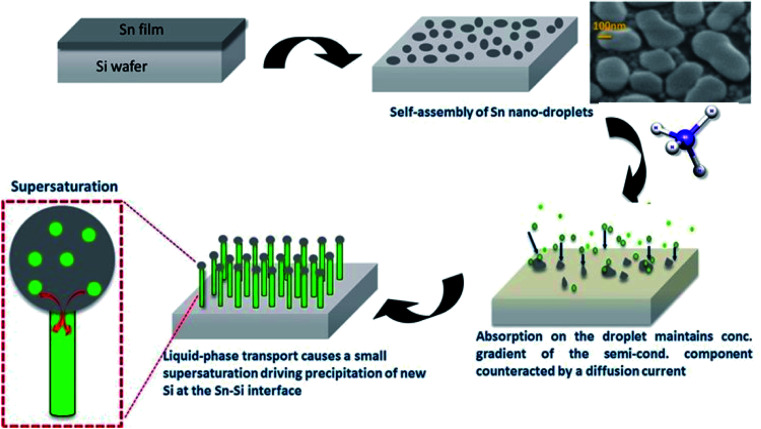
Schematic illustration of the growth of SiNWs *via* vapour–liquid–solid mechanism. Sn film coated substrate is exposed to H-plasma to obtain self-assembled Sn spherical nanoparticles. Si atoms are adsorbed and precipitated from Sn nanoparticles to obtain nanowires that are directly grown from the substrate (*i.e.* nanowires are electrically welded to the bottom conductive Cu sheet or any chosen substrate).

As illustrated in the schematic in Fig. 3, the kinetics of the VLS mechanism consist of four major steps:

(1) Mass transport of precursor in the gas phase.

(2) Chemical reactions at the vapour–liquid interface.

(3) Diffusion in the liquid phase.

(4) Incorporation and arrangement of atoms in a crystal lattice.^37^

Whilst studies on VLS SiNW growth are established using Sn as a catalyst,^45^ the application of such films as anode systems for Li-ion energy storage has not been comprehensively explored. The feasibility of growing these hybrid systems on alternative current collectors is tested here using flexible conductive carbon paper mesh as an advanced 3D electrode manufacturing approach. Flexible free-standing Li-ion batteries have been fabricated using carbon paper (with enhanced conductivity) as the current collector, specifically N-doped 3D porous carbon paper.^46^

## 2 Methods/experimental

### a. Plasma enhanced chemical vapour deposition & vapour–liquid–solid SiNW growth

Copper (Cu) foil and Cu mesh sheets were obtained from Dexmet Corporation and were cut to 30 × 30 mm and cleaned with a stream of nitrogen gas. Carbon paper Spectracarb 2050A-0850 was obtained from Toray through-plane resistivity of 18 mΩ cm^2^. Corning glass (Alkali Free Borosilicate-7059) and P-Silicon wafer (500 μm thickness, 1–20 ohm cm, 100 orientation) were cleaned using an RCA process and the native oxide on Si wafers was removed by immersing wafers in a buffered hydrofluoric (HF) acid solution for 10 seconds. The residual HF was removed by rinsing thoroughly with deionised water. The aforementioned substrates (Cu foil, Cu mesh, glass, carbon paper and Si wafer) were loaded in a thermal evaporator (Edwards AUTO 306) for deposition of 100 nm (mass thickness) thin film of tin (99.999% purity) at 10 Å s^−1^ at 8 × 10^−7^ mbar base pressure.

Real-time thicknesses were monitored using a quartz microbalance. All substrates coated with tin on one side of the substrates were loaded into a capacitive-coupled RF-PECVD (Radio Frequency-Plasma Enhanced Chemical Vapour Deposition) chamber (Oxford PlasmaLab) and pumped down to 5 mtorr base pressure. The temperature was raised to 400 °C and maintained for 5 min followed by hydrogen plasma pre-treatment (hydrogen gas flow at 100 sccm, 500 mtorr chamber pressure and, 33 mW cm^−2^ RF power density) for 5 min. Without breaking the vacuum, 20 sccm of SiH_4_ gas was introduced to existing H-plasma to initiate the growth process and growth of silicon nanowires (SiNWs) continued for 15 min. Samples were removed after PECVD chamber was let to cool down to below 50 °C.

### b. X-ray diffraction analysis

In this work, Bruker–D2 phaser equipped with 1-D LYNXEYE detector with a resolution of ±0.02° is utilised for the investigation. All scans were performed by Cu anode to produce X-rays at 30 kV and 10 mA to generate monochromatic X-rays with 1.54056 Å wavelengths. Ni filter is used to remove K_beta_ X-rays, thereby only K_alpha_ is incident over the sample. SiNWs grown on glass substrates from a tin catalyst with mass thickness 100 nm is used for investigating structural signature/phase identification.

### c. Electrochemical characterisation

All films were tested in Hohsen 2032 coin cells *vs.* a lithium foil counter electrode. The separator is PP/PE/PP microporous trilayer membrane (Celgard 2325) and the electrolyte is RD265 (PuriEL, SoulBrain, US) and is composed of EC, EMC, FEC and VC. The cell was cycled galvanostatically using a BioLogic VMP3 potentiostat with a low current module (10 nA limit). Comparison electrodes based on powder Si and Sn hybrids were fabricated using 50 : 50 active mass ratios (Elkem BV and Sigma Aldrich respectively) in mass proportions of 70 : 16 : 10 of active material : polyacrylic acid (450 k Sigma Aldrich) : Super-P (Timcal). Both cells used the same electrolyte solvent and charge–discharge cycling parameters.

### d. Scanning electron microscopy and focused ion beam cross-section preparation

**Fig. 4:**
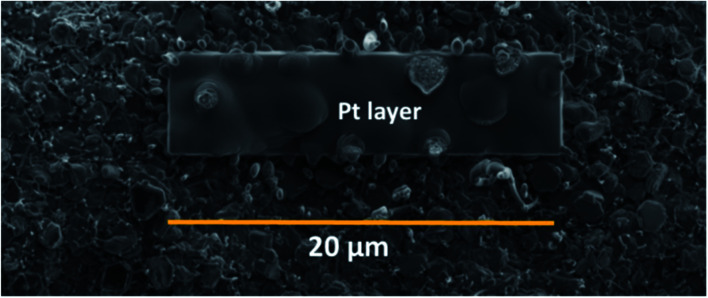
Sputtered coated Pt over the surface of SiNW–Sn film section for FIB-sectioning.

SEM images were obtained using a Carl Zeiss Sigma Ultra microscope using a working distance of 2–7 mm and accelerating voltage of 2 kV. Cross-section analysis was performed using a 1 nA current at 30 kV. For the cross-section analysis Pt was deposited (Fig. 4) to mitigate beam damage to the films. Different currents were used to dig the trench, ranging from 50, then 30, 7 and final finishing with a 1 nA current, all at 30 kV accelerating voltage.

## 3 Results and discussion

**Fig. 5:**
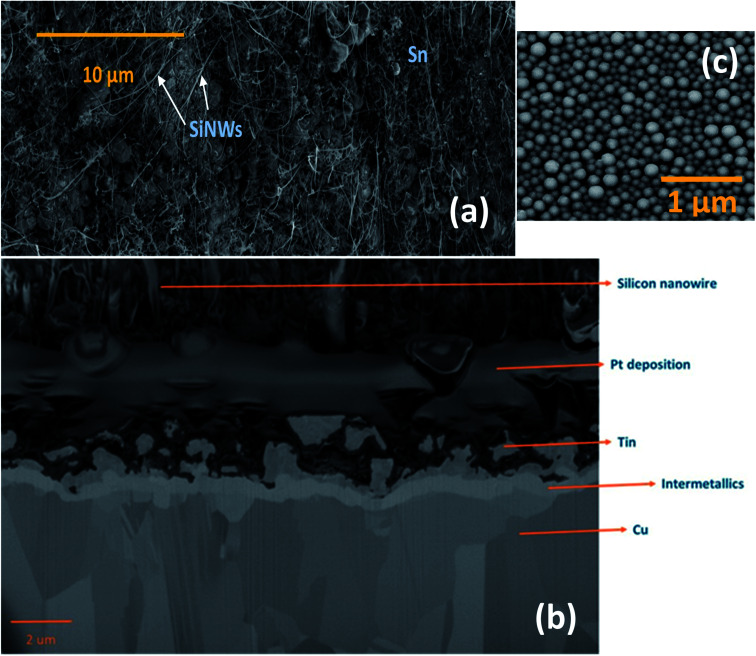
(a) Morphology of vapour deposited Sn–SiNWs film (top-view). (b) FIB cross-section of SiNW–Sn hybrid film deposited over copper foil. (c) Self-assembled spherical Sn catalyst nanoparticles layer.

The morphology and vertical cross-section microstructure of the vapour deposited Sn–SiNWs film is shown in Fig. 5(a) and (b). The Pt layer seen in the cross-section Fig. 5(b) is used to protect the sample from any damage induced by the ion beam. Identification of the phase composition of the intermetallic Cu–Sn layer by X-ray diffraction was challenging due to the thickness of the films making detection of the intermetallics very difficult. The Sn catalyst layer in Fig. 5(c) deposited prior to any Si alloying and precipitation, can be seen to appear as quite a uniformly self-assembled series of spherical deposits that are crystalline. The catalyst is subsequently treated under a H-plasma before the growth of SiNWs following introduction of the silane gas.

The assembly of layered thin films relies on interfacial integrity has been found to have a profound effect on the resulting function.^47^ The formation of intermetallics between Si, Sn and the underlying Cu substrate may significantly affect the stability of the interface – this could promote adhesion quality of the active anode film and warrants deeper investigation beyond the experimental scope of this investigation.

**Fig. 6:**
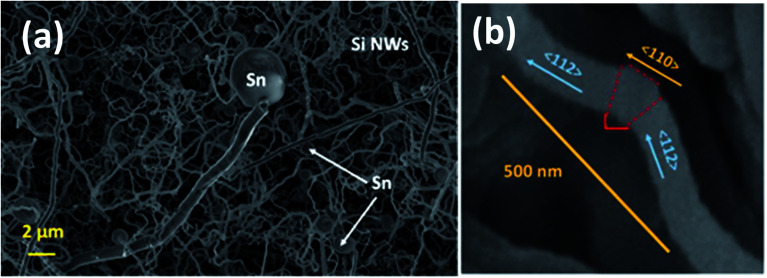
SEM image of Si–Sn showing (a) the Sn catalyst particles at the tip of SiNWs grown on a copper foil substrate and (b) single SiNW with angular kinks with crystalline phase identification of twinning regions.

The SiNWs have a very high aspect ratio, as can be seen in Fig. 6(a), with diameters typically around 100–150 nm and lengths of up to 10 μm, incorporating a significant mass of SiO_2_ shell. This cannot be directly or very easily quantified as the nanowires are combined with a Sn layer that will have its own associated oxide layer. With higher magnification it is evident that many of the NWs have kinks in their 2D morphology as shown in Fig. 6(a). The zig-zag morphology found in some of the nanowires is due to stacking-fault energy of Si (50 mJ m^−2^) – this generates twins during crystallisation^35^ as shown in Fig. 6(b). Twin defects are a special type of grain boundary occurring commonly in many different minerals and are perpendicular to the growth direction of the nanowire. Likely crystalline parameters are illustrated: single crystalline wires are connected by a 120° angle joint.

**Fig. 7:**
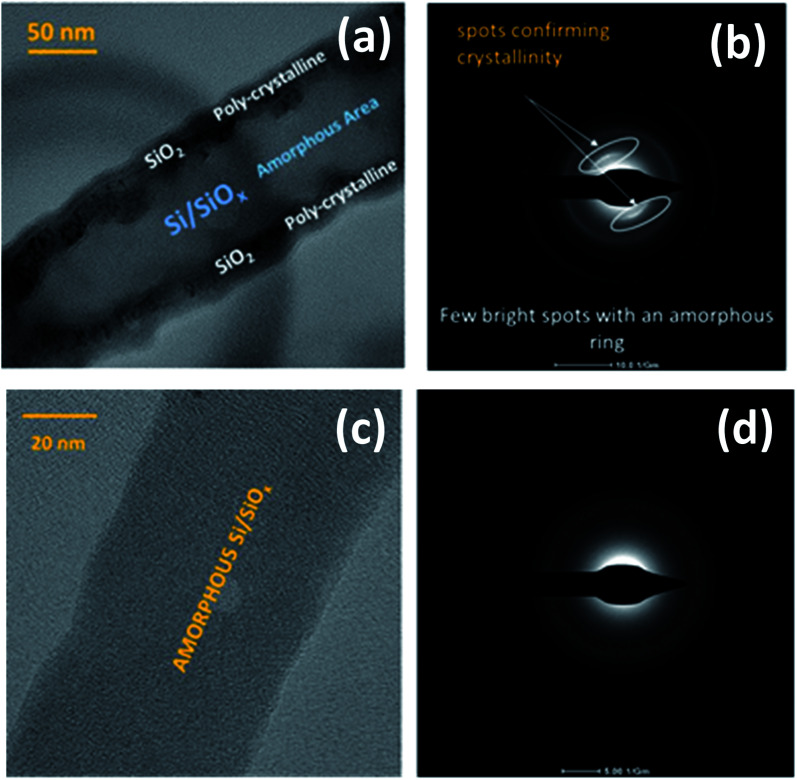
HRTEM and selected area electron diffraction imaging of SiNW with both crystalline and amorphous regions (a and b) and purely amorphous SiNW (c and d).

Two 〈112〉_c_ or 〈110〉_c_ vectors in a cubic crystal structure and two 〈11–20〉_h_ or 〈1–100〉_h_ vectors in a hexagonal structure can form such a 120° joint when rotating about the 〈111〉_c_ and 〈0001〉_h_ zone axes respectively.^48^ From the selected area electron diffraction (SAED) patterns in Fig. 7(a) and (b) there is evidence of both crystalline and amorphous phases within the hybrid films deposited in this work. This will have implications in the lithiation behaviour of the anode active materials during charging, this being attributable to charge transfer kinetics in amorphous – compared with crystalline – silicon.^49^ The complex two-phase lithiation of crystalline Si, subsequent lithiation phases that evolve in amorphous Si^50^ and the recrystallization of Si^51^ has received much attention and are still yet some way from being definitively concluded.

**Fig. 8:**
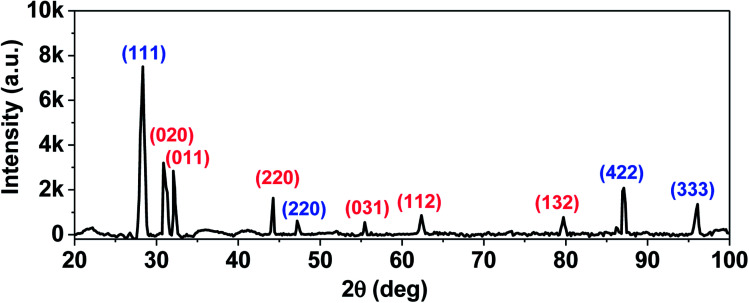
X-ray Diffraction pattern of Sn–SiNW film. Characteristic peaks that correspond to diffraction planes of Si (blue) and Sn (red) are highlighted.

The crystallinity of these films are further analysed by XRD as shown in Fig. 8. Characteristic peaks that correspond to diffraction planes of Si (blue) and Sn (red) are identified. The diffraction patterns of the deposited composite is purely composed of elemental state of Si and Sn. Similar patterns corresponding to pure elements have been observed for Si–Sn composites.^52^ The diffraction peak of Si indicates the cubic phase of SiNWs. These nanowires grown with tin at temperatures used here show increased lattice constants that is attributed to the bending of SiNWs, kinks in the nanowire and incorporation of Sn in the nanowire during the low temperature growth process. The lattice constant of tin is approximately 20% larger than silicon.^53^ The tetragonal Sn phase structure is evidenced by the peaks appearing at: 30.6°, 32.0°, 43.9°, 55.3°, 62.5° and 79.5° (JCPDS card no. 04-0673).

**Fig. 9:**
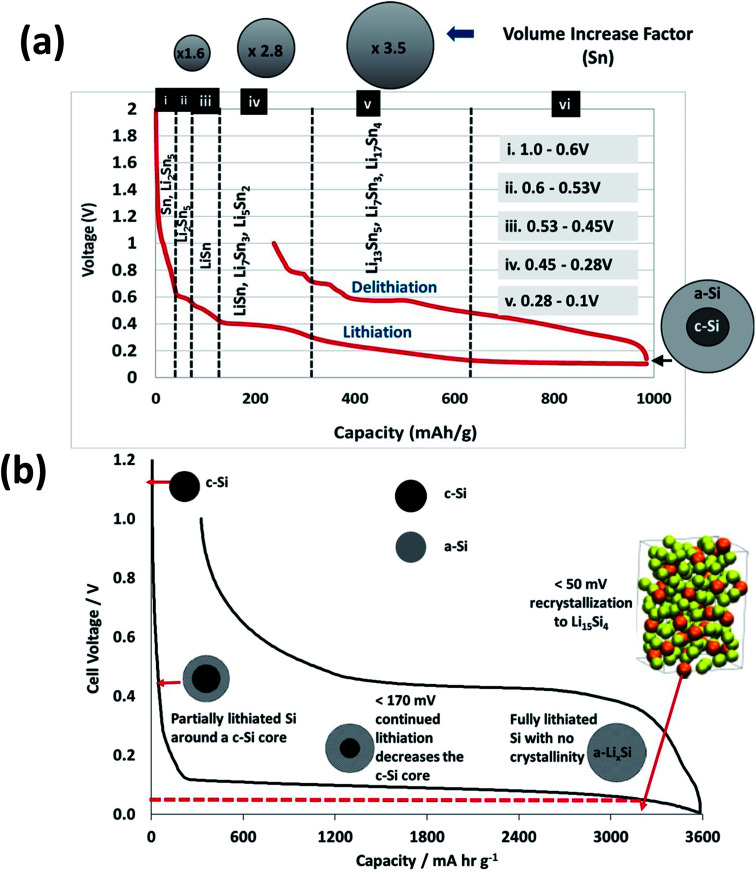
Voltage–capacity plots for the formation cycle showing key lithiation plateaus and associated voltage ranges (a) SnSi powder hybrid anodes (b) Si powder anodes lithiated to full capacity. NB schematic particles are not intended to be illustrative of scale.

Fig. 9(a) shows the 1^st^ cycle (formation) for the lithiation and delithiation to 1 V. Five different regions of varying slope can be identified for the lithiation (charging) reactions. These regions correspond to the evolution of a series of lithiated tin and silicon phases beginning with Li_2_Sn_5_ and ending with formation of Li_17_Sn_4_ for the Sn component of the active mass of the hybrid. In these films, interpreting the SiNW lithiation species as a function of voltage is somewhat more complicated compared with typical voltage profiles of the lithiation of bulk crystalline Si, as there are fewer defined voltage-specific plateaus.

Fig. 9(b) has more of a gradual slope rather than a series of plateaus, as is usual in the case with Sn lithiation voltage profiles. Additionally as the SiNWs are a combination of crystalline and amorphous phases, there will be less transition from crystalline-to-amorphous phase. In Fig. 9(b) the initial lithiation of crystalline bulk silicon results in a low voltage plateau corresponding to a two-phase region in which lithiated amorphous silicon is formed (a-Li_*x*_Si).^54,55^ Limiting the capacity to around 1000 mA h g^−1^ will avoid the deleterious volume expansion, which occurs when the highest lithiated Si species Li_15_Si_4_ is formed through recrystallisation. This phase formation is associated with an entire amorphous to crystalline transformation, whose onset occurs at around 60 mV and continues when the voltage < 50 mV. Such a phase transition can result in high internal stresses, leading to particle fracture and resulting capacity fade – a relationship has been suggested between film delamination and increased Li_15_Si_4_ formation.^56^ This is attributable to the Li_15_Si_4_ crystallization introducing grain boundaries which can in turn cause crack formation to propagate during delithiation, which may result in delamination.

Therefore, if the amorphous phase is maintained by remaining above 50 mV the electrode can in turn retain some structural stability. This is because conventional electrode manufacturing generates electrode architectures (microstructures) that cannot accommodate particle volume expansion to 280 vol% whilst retaining the cohesive, composite integrity of the as manufactured electrode coating. Obrovac states that the partial lithiation of crystalline Si results in particles composed of lithiated amorphous Si and completely non-lithiated crystalline Si, as shown schematically at the maximum capacity attained in Fig. 9.^54^

The reversible lithiation characteristics of amorphous silicon have been shown to retain longer structural stability than the crystalline material, attributable to increased tolerance to intrinsic stress and strain.^49^ Specifically the amorphous advantage lies in the ability to facilitate the isotropic stress and strain moments during the lithiation and delithiation processes.^49^

**Fig. 10:**
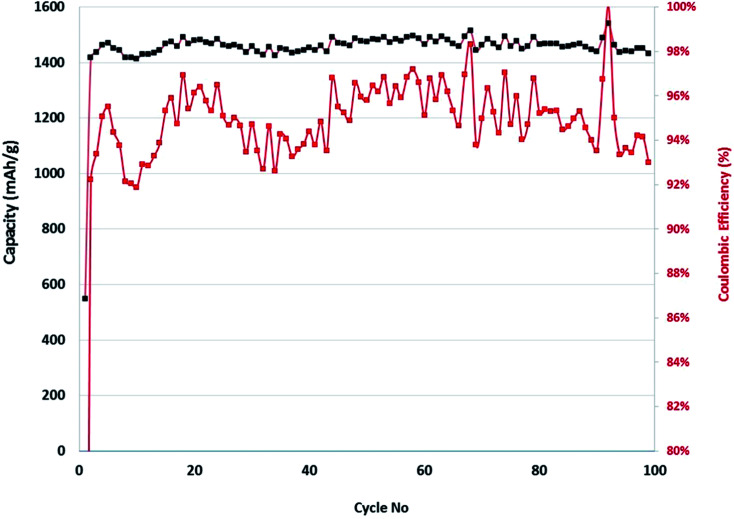
Discharge capacity & columbic efficiency *vs.* cycle number for Sn–SiNWs anode *vs.* Li/Li^+^.

The Sn/Si nanowire hybrid electrode structures (Region ii in Fig. 9a) showed the tin plateaus during the first part of lithiation, and then the silicon lithiation pseudo equilibrium beyond 500 mA h g^−1^. The plateau voltage was higher than typically observed for pure silicon electrodes (see Fig. 10). There was a large first cycle loss, and no observable Sn plateaus during delithiation. Part of this was due to the high surface area of the silicon nanowires. However, the absence of the tin voltage features suggests that some of the lithiated tin particles may have become isolated during the silicon expansion and contraction.

The Sn/Si particle electrode (Region iii in Fig. 9a) showed the tin voltage plateaus during delithiation, but not during lithiation, when they were “smeared” out and dominated by the voltage curve where the silicon is undergoing progressive lithiation. The electrode resistance was higher than the nanowire hybrid, due to the reduced number of contact points within the electrode structure. The voltage decreased during the silicon pseudo-equilibrium stage, since the decrease in silicon pseudo OCV was not matched by the usual decrease in resistance.

The discharge capacity as a function of cycle number is shown in 10 and there is a relatively good cycling performance to 100 cycles. However, this electrochemical testing was performed in half-cells against Li foil and this can compensate for the low columbic efficiency (CE) by supplying excessive Li ions as needed (which would not be the case in a full cell that has a finite Li inventory). In commercial cells to achieve industry-relevant cycling performance the CE needs to be ≥99.93% to achieve a target number of charge–discharge cycles (at least 300) before it reaches 80% capacity retention.

**Fig. 11:**
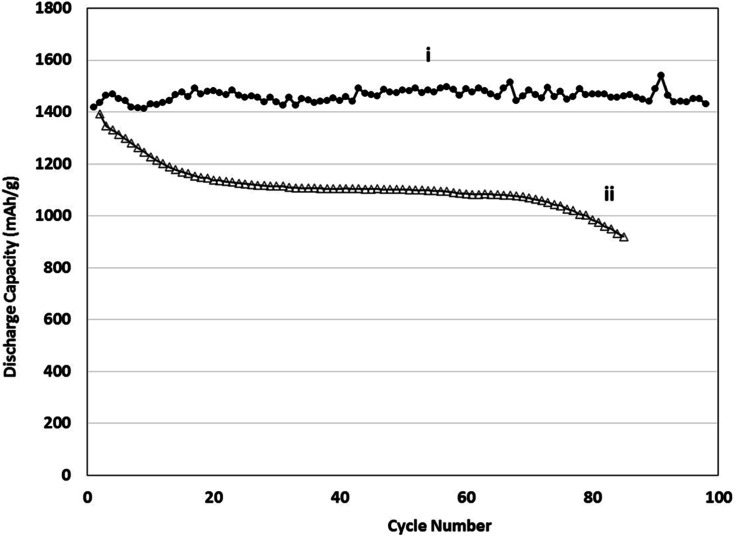
Discharge capacity *vs.* cycle number (i) PVD deposited Si–Sn electrodes, (ii) Pwd coated Si–Sn hybrid anodes *vs.* Li/Li^+^.

The electrochemical performance of physical vapour deposited (PVD) Sn–Si films was compared with conventional anodes containing powdered (Pwd) Sn–Si hybrid films as outlined in Fig. 11. The anode in (ii) was formulated by conventional means and comprised a polyacrylic acid binder with conductive carbon additives, consistent with conventional electrode manufacturing approaches. The powder hybrid system shows clear accelerated capacity fade rate beyond 80 cycles. The electrode microstructures of the two systems compared are entirely different on several levels, in terms of the electrode film composition and microstructure. Electrodes generated using a conventional composite film deposition approach are often susceptible to delamination phenomena, which result in progressive capacity fade.^57,58^ This can be attributable to the adhesive efficacy of the polymer binder fraction of the composite, often influenced by the polymer's functional groups.^59^ The growth of films of active masses directly from a current collector represents an alternative route to mitigating this effect. This comparative test was carried out to establish whether there is any clear performance benefit to generating a morphologically distinctive, less dense active mass, polymer-free film without the need for conductive additives.

**Fig. 12:**
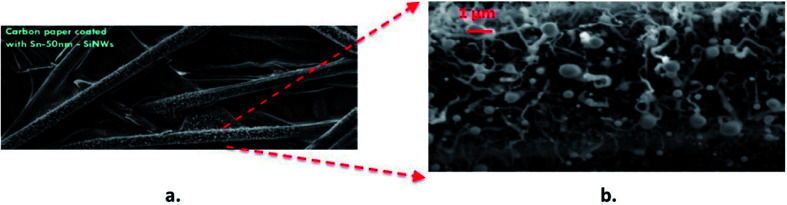
SEM images of Sn–Si hybrid nanowires grown on carbon cloth at (a) low magnification and (b) higher magnification down to low micron scale.

Fig. 12 shows low and high resolution images of VLS-grown SnSi NWs grown on conductive carbon paper, presenting exciting possibilities to generate flexible free-standing electrodes incorporating 3D current collector substrates. The electrochemical characterisation of these electrodes will be presented elsewhere in a separate study following the further optimisation of this film growth. nSi–C composites have been fabricated by ultra-sonication and positive-pressure filtration^60^ and were shown to stably cycle for a few hundred cycles. The development of flexible electrodes using carbon substrates has the potential to offer excellent thermal, electrical and mechanical properties – especially in applications that require the need to endure cyclical mechanical deformation.

## 4 Conclusion

A relatively low temperature deposition technique was shown to generate a binary anode system composed of both crystalline and amorphous SiNWs, a dense Sn layer and some interfacial copper–tin intermetallic phases. Sn has been shown to be an interesting catalyst for SiNW growth as it is isoelectric with Si thus a neutral “impurity”. PECVD of Si in combination with Sn catalyst seeds opens up exciting possibilities at low temperatures. This is facilitated by the dissociation of the precursor gas in a low power plasma, allowing lowering of the substrate temperature whilst maintaining high SiNW growth rate. This non-optimised film was able to endure reversible lithiation for 100 cycles at nearly four times the maximum capacity of graphite. Conventional powder-binder-additive containing electrodes did not achieve this level of performance in a direct cycle life comparison.

Subsequent work will follow on the systematic investigation into optimal SiNW density, morphology, crystallinity, film thickness parameters and alternative 3D current collectors. Also to be investigated is how to control and optimise deposition parameters on a variety of flexible, conductive substrates. Additional optimisation extending into ternary chemistries may be a possible route to generating more stable amorphous alloy films/metastable structures capable of efficient, reversible lithiation for several hundreds of charge–discharge cycles.

Alternative manufacturing routes such as low temperature PECVD do not require indirect materials or energy-intensive processing, and could be a key enabler generating cheaper and long lasting electrode architectures. Their ability to deposit onto three-dimensional structures could present opportunities beyond the capability of conventional coating techniques. This can enable the use of metallic foam or carbon-based current collectors in batteries for very high rate applications. The potential for use in flexible or wearable energy storage could also become increasingly possible by this approach. Further developing the capability to generate wearable energy storage is of much practical interest although it is far from being at a level ready for commercial exploitation. PECVD is an established, scalable technology that is well-suited to depositing functional semiconductor coatings. Our strategy presents new possibilities to explore and better understand the approaches to designing and understanding new electrode materials and microstructures, to correlate structure with performance, and how to better optimise energy storage components.

## Conflicts of interest

There are no conflicts to declare.

## Supplementary Material
